# Bio-Printed PCL Tracheal Graft in a Large Animal Model: Reproducible Short-Segment Regeneration and Preliminary Upgraded Long-Segment Reconstruction

**DOI:** 10.3390/bioengineering13030324

**Published:** 2026-03-12

**Authors:** Sen-Ei Shai, Yi-Ling Lai, Yi-Wen Hung, Chi-Wei Hsieh, Yun-Jie Hung, Kuo-Chih Su, Chun-Hsiang Wang, Chia-Ching Wu, Shih-Chieh Hung

**Affiliations:** 1 Department of Surgery, Division of Thoracic Surgery, Taichung Veterans General Hospital, Taichung 407219, Taiwan; windjay77@hotmail.com; 2 School of Medicine, National Yang Ming Chiao Tung University, Taipei 112304, Taiwan; 3 Department of Post-Baccalaureate Medicine, College of Medicine, National Chung Hsing University, Taichung 402202, Taiwan; 4 Department of Applied Chemistry, National Chi Nan University, Nantou 545301, Taiwan; 5 Terry Fox Cancer Research Laboratory, Translational Medicine Research Center, China Medical University Hospital, Taichung 404327, Taiwan; hongiw@yahoo.com.tw; 6 School of Medicine, National Cheng Kung University, Tainan 701401, Taiwan; peter100yahoo@gmail.com (C.-W.H.); joshccwu@mail.ncku.edu.tw (C.-C.W.); 7 Department of Medical Research, Taichung Veterans General Hospital, Taichung 407219, Taiwan; yjhung@vghtc.gov.tw; 8 Institute of Biomedical Sciences, National Chung Hsing University, Taichung 402204, Taiwan; 9 Three Dimensional Printing Research and Development Group, Department of Medical Research, Taichung Veterans General Hospital, Taichung 407219, Taiwan; kcsu@vghtc.gov.tw (K.-C.S.); wangch@vghtc.gov.tw (C.-H.W.); 10 Department of Cell Biology and Anatomy, College of Medicine, National Cheng Kung University, Tainan 701401, Taiwan; 11 Integrative Stem Cell Center, China Medical University Hospital, Taichung 404327, Taiwan; hung3340@gmail.com; 12 Institute of New Drug Development, China Medical University, Taichung 404328, Taiwan

**Keywords:** three-dimensional bioprinting, tracheal tissue engineering, polycaprolactone (PCL) graft, large-animal model, airway regeneration

## Abstract

Three-dimensional (3D) bioprinting provides new options for airway reconstruction by enabling the fabrication of customizable, biodegradable scaffolds designed to support in situ tissue regeneration. Building on our established large-animal platform, in which two cm bioprinted tracheal grafts combined with refined surgical techniques and adjunctive laser intervention have achieved long-term survival exceeding three months, the present study aims to explore long-segment (≥four cm) tracheal transplantation. We evaluated the fabrication feasibility and regeneration patterns of extrusion-based 3D bioprinted polycaprolactone (PCL) tracheal grafts in a porcine model. The grafts were implanted via end-to-end anastomosis with adjunctive mechanical stabilization and followed by serial bronchoscopic surveillance, gross examination, and histological analysis. The two cm PCL tracheal grafts achieved reproducible survival exceeding three months when combined with refined surgical techniques, structured postoperative airway management, and optimized wound coverage. Histological analysis revealed multi-lineage tissue formation—including cartilage, muscle, glands, and epithelium—was observed. Cartilage regeneration followed a staged maturation process, compared to epithelial regeneration, although continuous by 12 weeks, remained developmentally immature. A single long-segment transplantation was explored in a single preliminary case, providing an initial technical observation of feasibility; however, definitive conclusions regarding long-term survival or regeneration cannot be drawn. These findings further characterize regenerative responses in a large-animal model and highlight critical translational barriers—fabrication constraints, airway biomechanics, and delayed epithelial maturation—that require systematic investigation before long-segment tracheal reconstruction can advance toward clinical application.

## 1. Introduction

Tracheal transplantation remains one of the most challenging areas in organ replacement surgery and has not yet achieved broadly accepted clinical success. For patients with long-segment tracheal stenosis, a successful tracheal substitute would represent a definitive and potentially curative treatment. However, previous attempts at direct human tracheal transplantation have resulted in major scientific and ethical controversies, leading to a global consensus that any artificial trachea or tracheal transplantation strategy must first demonstrate safety and feasibility in large-animal models before clinical application [1,2,3,4,5,6,7]. The challenges associated with large-animal tracheal transplantation far exceed those encountered in conventional tissue engineering studies, encompassing respiratory biomechanics, persistent negative intrathoracic pressure, infection risk, and substantial anastomotic tension. Although circumferential resection with end-to-end anastomosis is considered the standard surgical approach, its application is constrained by resection length and technical limitations. Consequently, alternative strategies for long-segment airway reconstruction continue to be investigated.

The structural and biomechanical characteristics of the trachea contribute substantially to the difficulty of airway replacement. The adult trachea measures approximately 11.8 cm in length and typically consists of 18–22 cartilage rings, with marked interindividual variation influenced by age, body habitus, and spinal curvature [8]. The airway comprises rigid C-shaped cartilage anteriorly and a compliant posterior membranous wall that dynamically adapts to respiration, coughing, and changes in intrathoracic pressure [9,10,11]. Airway geometry and compliance may be further altered by pathological conditions such as chronic obstructive pulmonary disease, tracheobronchial malacia, and tracheobronchopathia osteochondroplastica [11]. These anatomical and biomechanical factors limit the performance of existing substitutes—including prostheses, autografts, allografts, xenogeneic scaffolds, and tissue-engineered tracheas—which continue to face challenges related to donor availability, immunological compatibility, mechanical mismatch, and long-term functional stability [1,2,3,4,5,6,7,12]. Long-segment tracheal stenosis (>six cm), therefore, remains difficult to address, and postoperative restenosis is a frequent cause of failure, particularly in large-animal and translational studies [3,13,14,15,16,17,18,19].

Tissue engineering has been developed as an approach to fabricate functional tissues through the integration of biomaterial scaffolds, cells, and bioactive signals, with the goal of restoring or replacing damaged organs [20]. In parallel, advances in three-dimensional (3D) bioprinting have enabled the fabrication of tissue-engineered tracheal constructs with complex and customizable architectures that are difficult to achieve using conventional manufacturing techniques [21,22,23]. These developments support patient-specific design and provide new opportunities for airway reconstruction. Regenerative medicine further emphasizes in situ tissue engineering, in which implanted scaffolds leverage the host’s intrinsic regenerative capacity and function within the physiological environment of the airway [24,25,26,27]. However, evaluation of such strategies critically depends on large-animal models, as small-animal systems cannot adequately replicate human airway biomechanics, negative intrathoracic pressure, airflow dynamics, or postoperative infection risk. To date, most reported long-segment tracheal substitutes have demonstrated limited survival, frequently failing to exceed 60 days in large-animal models [3,17,18,19,28].

Since 2016, our group has systematically investigated 3D-printed tissue-engineered tracheal grafts based on polycaprolactone (PCL), fabricated using extrusion-based bioprinting while avoiding organic solvents that may adversely affect biocompatibility [28,29,30,31,32]. Using histological and immunohistochemical analyses, we previously demonstrated the in situ regeneration of multiple tissue components, including cartilage, epithelium, muscle, adipose tissue, and glandular structures, potentially originating from mesenchymal stem cells [29,30]. We further characterized temporal changes in protein expression and their co-occurrence with epithelial differentiation, subepithelial fibrosis, airway muscle remodeling, and microvascular adaptation, processes that collectively influence extracellular matrix remodeling and long-term graft integration [30,33]. Despite these advances, challenges related to airway patency and postoperative stability persist. Granulation tissue formation remains a common cause of luminal narrowing, and infection—particularly involving Gram-negative organisms—continues to compromise graft performance. In addition, tracheal grafts implanted in large-animal models must withstand substantial anastomotic tension and sustained negative intrathoracic pressure [28,31,34].

Building on our established large-animal platform, in which two cm bioprinted tracheal grafts have demonstrated reproducible regeneration and survival exceeding three months, the present study primarily investigates this validated short-segment platform while exploring the technical feasibility of extending the approach toward longer graft configurations. Specifically, we examined the surgical feasibility of a four cm graft implantation as a preliminary exploratory extension toward long-segment reconstruction in a porcine model. Given the complexity of long-segment airway reconstruction, this experiment is intended as an exploratory feasibility observation rather than definitive validation of long-segment efficacy. Through this investigation, we aim to further characterize the translational challenges associated with long-segment tracheal reconstruction in a large animal setting.

## 2. Materials and Methods

### 2.1. Study Design and Experimental Overview

This study was designed to evaluate the surgical feasibility and regenerative response following the transplantation of bio-printed, biodegradable PCL tracheal grafts in a large animal model. Building upon our established porcine platform, in which two cm (20 mm × 20 mm × 1.5 mm) circumferential PCL tracheal grafts combined with innovative surgical techniques and adjunctive laser intervention consistently achieved survival beyond 90 days [28,31], the current work primarily investigates this validated short-segment platform while exploring long-segment tracheal replacement (≥four cm, ≥40 mm × 20 mm × 1.5 mm). The experimental framework integrates graft fabrication, surgical implantation, postoperative airway management, and histological assessment, following methodologies previously established in our large animal studies.

It should be noted that long-segment transplantation was performed as a single exploratory feasibility case (*n* = 1) to evaluate surgical reproducibility and early postoperative airway status, rather than establish definitive long-term survival or regenerative outcomes.

### 2.2. Design and Fabrication of Bio-Printed PCL Tracheal Grafts

Tracheal grafts were designed using computer-aided design (CAD) software (Cellink HeartWare 2.4) based on the high-resolution digitization of native porcine tracheas, as previously described [31]. The finalized designs were converted into standard STL files and subsequently processed into G-code for extrusion-based bioprinting. Medical-grade PCL filament ((CAPROLACTONE™, Lattice Services, Paris, France) was used as the primary scaffold material due to its established biocompatibility, mechanical stability, and solvent-free processing characteristics.

All grafts were fabricated using a CELLINK BIO X bioprinter via fused deposition modeling. Printing parameters—including nozzle diameter, extrusion pressure, printing speed, layer thickness, and infill density—were optimized based on our prior work to achieve a wall thickness of approximately 1.5 mm and an infill density of 25%. This configuration was selected to provide adequate structural flexibility while maintaining sufficient radial stability for implantation. Mechanical characterization of the fabricated grafts, including compressive resistance and elastic recovery testing using a JSV-H1000 system (Japan Instrumentation System Co., Nara, Japan), was performed prior to implantation to verify structural stability of the scaffold under simulated loading conditions. Direct quantitative comparison with native tracheal mechanical properties was not performed in the present study. Detailed quantitative datasets are part of an ongoing engineering-focused study and are, therefore, not fully disclosed in this report. The graft architecture incorporated multiple distribution holes to facilitate uniform nutrient diffusion, tissue ingrowth, and integration with host tissue. Following fabrication, grafts were sterilized using ethanol and ultraviolet irradiation according to established protocols [31]. After sterilization, graft sterility was verified by incubation in a cell culture environment to confirm the absence of contamination prior to implantation. This sterilization approach was applied for preclinical experimental use; clinical-grade sterilization methods (e.g., gamma irradiation or ethylene oxide) would be required for future translational application.

### 2.3. Experimental Animals and Ethical Approval

A total of six porcine models were included in this study, encompassing survival outcomes and postoperative clinical observations. The cohort consisted of three LYD porcines and three Lanyu porcines. The transition from LYD to Lanyu pigs occurred during different experimental phases and was not designed as a controlled comparative study between breeds. All animals were approximately six months of age at the time of surgery, with body weights ranging from 30 to 40 kg. All experimental protocols were reviewed and approved by the Institutional Animal Care and Use Committee of Taichung Veterans General Hospital (IACUC approval No. La-1142201, approved on 6 May 2025) and were conducted in strict accordance with national and international guidelines for animal welfare. The limited sample size reflects the technical complexity of large animal tracheal transplantation and the exploratory nature of the present preclinical research.

### 2.4. Surgical Procedure for Tracheal Transplantation

All surgical procedures were performed under general anesthesia induced with intramuscular Zoletil 50 (8 mg/kg) and maintained with inhaled isoflurane (4%). Animals were positioned supine, and the cervical trachea was exposed via a sterile midline incision. In the standard procedure, a two cm circumferential segment of the cervical trachea was resected and reconstructed using a bioprinted PCL tracheal graft. End-to-end anastomosis was performed using non-absorbable sutures, following a previously described technique [34]. To reduce anastomotic tension, traction sutures were placed at the proximal and distal tracheal ends, and the graft was anchored to the bilateral strap muscles [31]. For wound coverage and soft tissue management, collagen-based biomaterials (Life Spring Co., Ltd., Taichung, Taiwan) were applied intraoperatively. These included a collagen extracellular membrane derived from small intestinal submucosa (SIS), a collagen matrix sheet, and a collagen matrix cube. The materials were placed over the surgical field and surrounding soft tissue after graft implantation, replacing the use of conventional surgical drainage tubes. The introduction of collagen-based wound coverage occurred during later experimental phases and was not intended as a controlled comparison of wound management strategies. Long-segment (≥4 cm) tracheal graft implantation was performed using the same graft design, surgical exposure, anastomotic technique, and anchoring strategy. This procedure was conducted as an exploratory feasibility attempt to evaluate surgical handling and early airway stability.

### 2.5. Postoperative Care, Bronchoscopy, and Laser Intervention

Postoperative management included analgesia (diclofenac potassium), antibiotic prophylaxis (ampicillin or amoxicillin–clavulanate), and mucolytic therapy (bromhexine hydrochloride), administered orally under veterinary supervision. Animals were closely monitored for respiratory status, feeding behavior, and signs of infection. Repeated endoscopic evaluation (OlympusBF-260/CV-260/CLV-260, Yuan Yu Industry Co., Ltd., Taichung, Taiwan) allowed timely identification of intraluminal tissue overgrowth and facilitated targeted intervention before potential airway compromise developed. In cases of clinically significant granulation tissue causing luminal narrowing, argon plasma coagulation or laser ablation (FiAPCprobe 1500A/VIO200D, ERa Bioteq Enterprise Co., Ltd., Taipei, Taiwan) was applied. Postoperative airway interventions were performed according to standardized surveillance protocols established in our previous large animal studies [34].

### 2.6. Tissue Harvesting and Histological Analysis

At predefined endpoints or upon euthanasia, graft-associated tissues were harvested en bloc for gross and histological examination. Specimens were fixed in 10% neutral-buffered formalin, embedded in paraffin, and sectioned for routine and specialized staining. Hematoxylin and eosin (H&E) staining was used for general morphology, while Alcian blue and Safranin O/Fast Green staining were applied to assess glycosaminoglycan content and cartilage maturation. Immunohistochemical analyses targeting lineage-specific markers (Type II collagen and Sox9) were performed as previously described to evaluate chondrogenesis, epithelialization, glandogenesis, myogenesis, and microvascular development [35]. Quantitative histomorphometric criteria applied in earlier publications were used for interpretative consistency; however, the present study focuses primarily on descriptive characterization of regenerated tissues and evaluation of the long-segment setting rather than formal quantitative morphometric analysis.

### 2.7. Comparative Analysis with Native Tracheal Tissue

To contextualize regenerative tissue maturity, native tracheal specimens harvested from age-matched porcines were processed in parallel. Histological features of regenerated tissue were qualitatively compared with native tracheas to assess layer-specific organization, cartilage maturation, epithelial integrity, and glandular development, following analytical frameworks established in our prior work [35].

## 3. Results

### 3.1. Fabrication Feasibility of Long-Segment Bioprinted Tracheal Grafts

**Figure 1:**
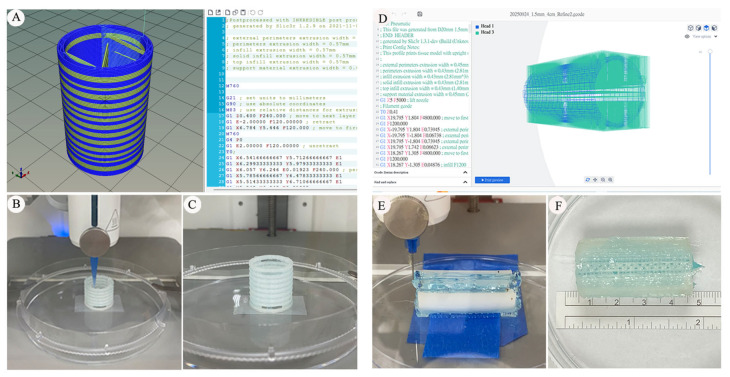
Fabrication feasibility and printing limitations of bioprinted tracheal grafts. (**A**) Computer-aided design (CAD) of a short-segment (two cm) tracheal graft using Cellink HeartWare software with customized G-code editing to define circumferential filament deposition and layer alignment. (**B**) Extrusion-based bioprinting of the two cm polycaprolactone (PCL) tracheal graft in a vertical orientation. (**C**) Completed two cm PCL tracheal graft demonstrating uniform wall thickness and stable lumen geometry. (**D**) Design of a long-segment (four cm) tracheal graft using DNA Studio 4 Vault software (CELLINK AB, Gothenburg, Sweden) with advanced G-code modification to accommodate increased graft length and internal architecture complexity. (**E**) Extrusion-based bioprinting of the four cm PCL tracheal graft, illustrating prolonged printing duration and increased demands on nozzle travel and layer stability. (**F**) Gross appearance of the fabricated four cm PCL tracheal graft. Although continuous fabrication was achievable, minor deviations in filament stacking and interlayer alignment were observed, reflecting reduced structural tolerance coincided with long-segment vertical printing.

The fabrication feasibility of bio-printed PCL tracheal grafts was first evaluated by comparing short-segment (two cm) and long-segment (four cm) designs using extrusion-based bioprinting. For the established artificial two cm graft, scaffold geometry was designed using customized CAD-based design with direct G-code editing, and printing paths were further refined by direct editing of G-code instructions (Figure 1A–C, left panel). This approach enabled controlled filament deposition and stable layer alignment, resulting in consistent tubular constructs with maintained lumen geometry. Two cm graft constructs were fabricated consistently under the selected printing parameters, demonstrating stable structural configuration in repeated prints.

In contrast, fabrication of long-segment (four cm) tracheal grafts posed additional technical challenges related to printer hardware constraints and construct stability. To address the increased design complexity, the four cm graft was modeled using customized CAD-based design with direct G-code editing, which allowed for the flexible manipulation of printing trajectories and internal architecture through G-code modification (Figure 1D–F). Continuous extrusion of a four cm tubular graft was achievable; however, vertical printing of constructs approaching or exceeding three cm introduced increasing mechanical and kinematic constraints of the bioprinter, including nozzle travel range, cumulative positioning error, and greater susceptibility to gravitational deformation during layer-by-layer deposition.

As graft length increased, minor deviations in filament stacking and interlayer alignment were occasionally observed, particularly in the upper segments of vertically printed constructs. Although fabrication remained technically achievable, these observations suggest reduced structural tolerance in longer constructs compared with shorter grafts. Furthermore, prolonged printing time for 4 cm grafts was associated with increased susceptibility to thermal fluctuation and filament sagging, underscoring the importance of optimized toolpath planning and support strategies for long-segment constructs.

Collectively, these observations indicate that while short-segment (two cm) PCL tracheal grafts can be fabricated consistently using the current printing configuration and direct G-code editing strategy [28]. In contrast, the fabrication of longer graft constructs (four cm) revealed practical engineering constraints associated with vertical build height and construct stability. These findings, therefore, represent qualitative technical observations that inform the subsequent evaluation of surgical feasibility and in vivo performance, rather than a quantitative assessment of fabrication accuracy.

### 3.2. Long-Term Survival Supported by Mechanical Stabilization and Postoperative Airway Management in the Established Two-cm Model

**Figure 2:**
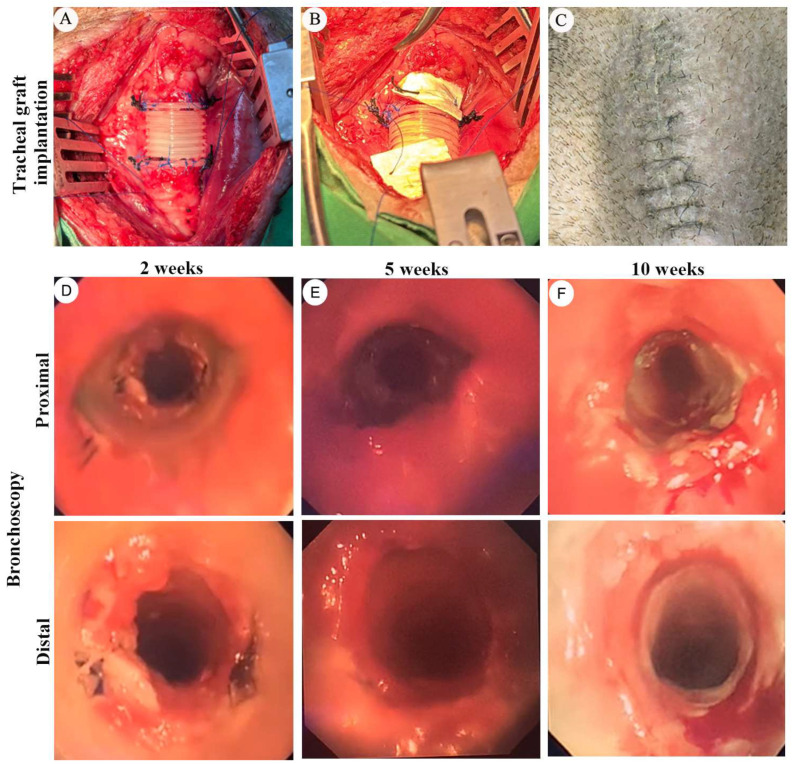
Long-term airway patency supported by mechanical stabilization and postoperative airway management in the established two cm tracheal graft model. (**A**–**C**) Intraoperative photographs showing circumferential transplantation of a two cm bio-printed PCL tracheal graft into the cervical trachea of a Lanyu porcine model. (**A**) The graft was implanted using end-to-end anastomosis and (**B**) reinforced anchoring to the strap muscles to reduce anastomotic tension and maintain axial airway alignment. (**C**) Postoperative wound healing of the neck at 10 weeks after implantation. (**D**–**F**) Serial bronchoscopic examinations of the grafted airway at two (**D**), five (**E**), and 10 (**F**) weeks after implantation. Bronchoscopic views from proximal and distal anastomotic sites demonstrate postoperative airway patency over time, with variable degrees of mucosal edema and granulation tissue formation during early follow up. Targeted endoscopic intervention was applied when necessary to maintain luminal patency.

In the established two cm tracheal graft model, animals were observed to survive beyond three months in those receiving integrated mechanical stabilization and structured postoperative airway management. Intraoperative findings confirmed successful circumferential implantation of the bioprinted PCL graft with secure end-to-end anastomosis to the native trachea (Figure 2A–C). Reinforced anchoring of the graft to the bilateral strap muscles helped maintain axial alignment of the airway and may reduce anastomotic stress under physiological respiratory motion.

Serial bronchoscopic examinations were performed to assess airway patency and luminal changes during follow-up. Early postoperative bronchoscopy revealed variable mucosal edema and granulation tissue formation at the anastomotic regions. When luminal compromise was identified, targeted endoscopic laser ablation was applied to restore airway caliber. Subsequent bronchoscopic evaluations generally demonstrated maintenance of a patent central airway during follow up, without evidence of graft collapse or displacement (Figure 2D–F).

Postoperative wound management strategies differed between experimental cohorts. In earlier experiments utilizing conventional surgical drainage tubes, postoperative wound infection occurred in a subset of animals. In animals managed without drainage tubes and treated with collagen-based wound coverage materials, no overt surgical site infection was observed during the observation period. Bronchoscopic observations in this group appeared to demonstrate relatively smoother luminal surfaces and fewer visible inflammatory changes during follow up. These observations represent descriptive findings only and were not derived from a controlled comparative design.

Overall, animals in the two cm tracheal graft model were able to maintain airway continuity during the observation period under a management framework that included mechanical stabilization, serial bronchoscopic surveillance, and postoperative infection and secretion control. These observations provide a large animal experimental platform that may support future exploration of extended graft configurations.

### 3.3. Spatial–Temporal Organization and Histological Maturation of Regenerated Tissues in the Two cm Tracheal Graft

**Figure 3:**
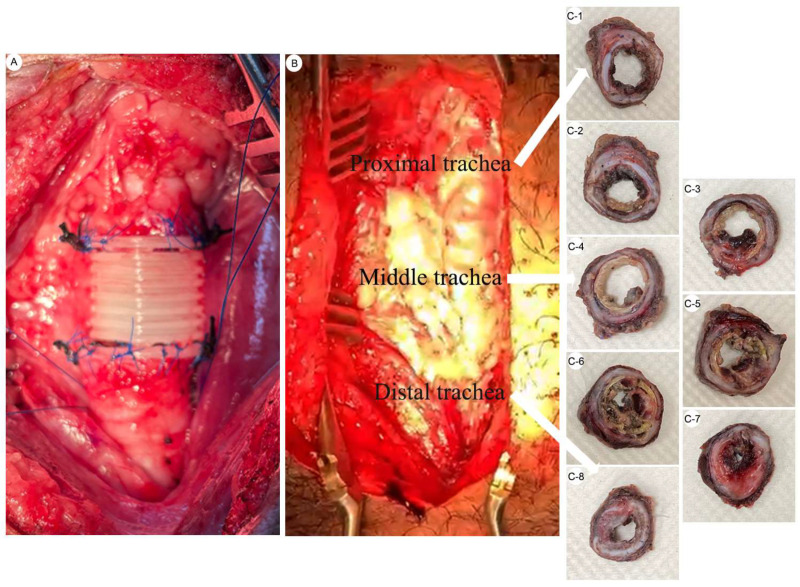
Spatial–temporal tissue regeneration within the established two cm tracheal graft at 12 weeks after implantation. (**A**) Intraoperative view showing circumferential implantation of the two cm bio-printed PCL tracheal graft with stable integration into the native trachea. (**B**) Gross surgical exposure illustrating the proximal, middle, and distal regions of the regenerated trachea at the time of tissue harvest, 12 weeks after implantation. (**C-1**–**C-8**) Representative cross-sectional specimens obtained along the graft length, from proximal to distal anastomosis, demonstrating spatial variation in luminal configuration and tissue coverage at 12 weeks after implantation. Arrows indicate the sequential order of specimen sampling from the proximal to the distal trachea.

**Figure 4:**
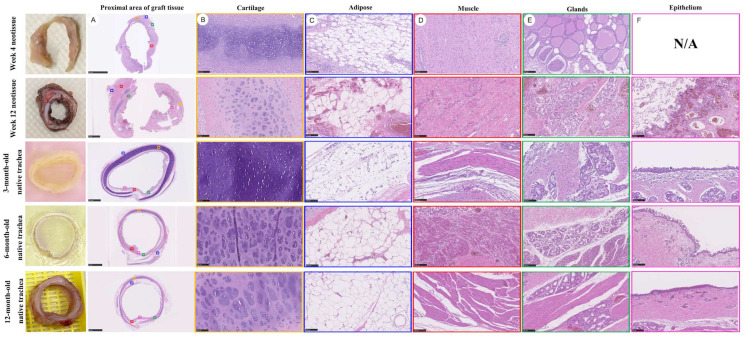
Histological comparison of regenerated tracheal tissue and native trachea at different stages of maturity. Representative hematoxylin and eosin (H&E)-stained sections showing tissue composition and organization in regenerated tracheal grafts and native trachea. Regenerated tissues at four and 12 weeks after implantation are compared with native tracheal specimens harvested from three-, six-, and 12-month-old animals. Colored boxes indicate regions of interest in the total-view images, and the corresponding magnified histological views are shown in the adjacent columns. Columns depict distinct tissue components (**A**), including cartilage (**B**), adipose tissue (**C**), muscle (**D**), glands (**E**), and epithelium (**F**). At 12 weeks after graft implantation, regenerated tissue demonstrates partial recapitulation of native tracheal architecture, with identifiable cartilage-like structures, muscle-like tissue, and epithelial coverage, although overall tissue organization and maturity remain less developed than those observed in age-matched native trachea. N/A: not applicable. Scale bars: 5 mm for (**A**); 100 µm for (**B**–**F**).

**Figure 5:**
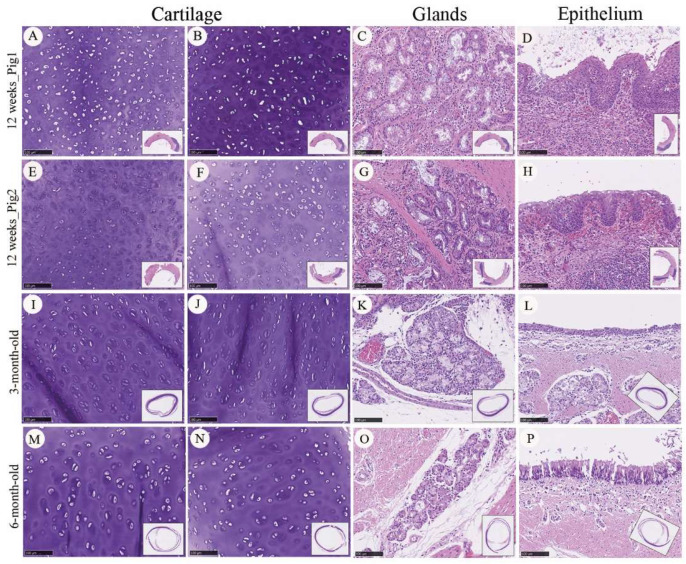
Histological comparison of regenerated and native tracheal tissues at different maturation stages. Representative hematoxylin and eosin (H&E)-stained sections showing cartilage, glandular, and epithelial components in regenerated tracheal tissue and native trachea. (**A**–**H**) Regenerated tracheal tissue harvested 12 weeks after implantation of the two cm PCL graft, demonstrating cartilage with relatively high cellular density (**A**,**B**), identifiable glandular clusters within the submucosal region (**C**,**G**), and continuous but irregular epithelial coverage along the luminal surface (**D**,**H**). (**I**–**L**) Native tracheal tissue from three-month-old animals, exhibiting more organized cartilage structure, glandular distribution, and epithelial stratification. (**M**–**P**) Native tracheal tissue from six-month-old animals, demonstrating further maturation with denser cartilage matrix, well-defined glandular architecture, and thicker, more organized epithelial lining. Scale bars: 100 µm.

**Figure 6:**
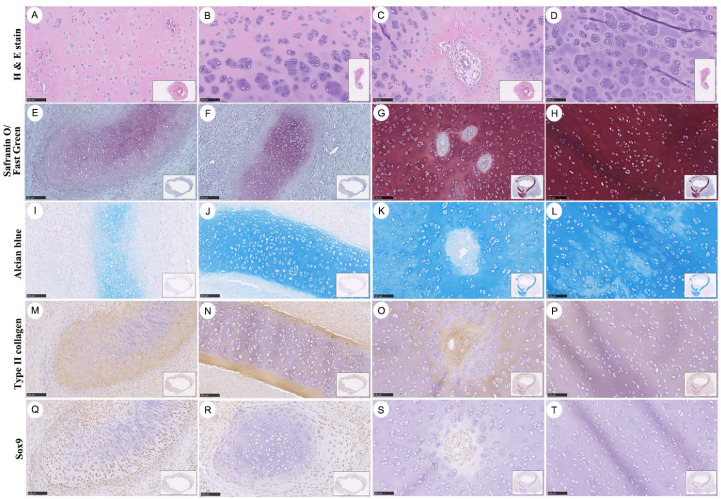
Staged cartilage regeneration within graft-associated neotissue. Representative histological and immunohistochemical analyses illustrating progressive stages of cartilage maturation within graft-associated neotissue. (**A**–**D**) H&E staining at 12 weeks after implantation showing early-stage cartilage with sparse chondrocyte-like cells and limited ECM deposition (**A**,**B**), intermediate-stage cartilage with focal matrix condensation and vascular canal (VC)-associated chondro-modulator structures (**C**), and later-stage cartilage with increased matrix density and more homogeneous architecture (**D**). (**E**–**H**) Safranin O/Fast Green staining demonstrating progressive enrichment of proteoglycan-rich cartilage matrix. (**I**–**L**) Alcian blue staining showing increasing glycosaminoglycan (GAG) deposition across maturation stages. (**M**–**P**) Type II collagen immunostaining revealing early and sustained expression throughout cartilage development. (**Q**–**T**) Sox9 immunostaining illustrating higher expression in early-stage cartilage with gradual reduction during maturation, consistent with staged chondrogenesis. Scale bars: 100 µm.

**Figure 7:**
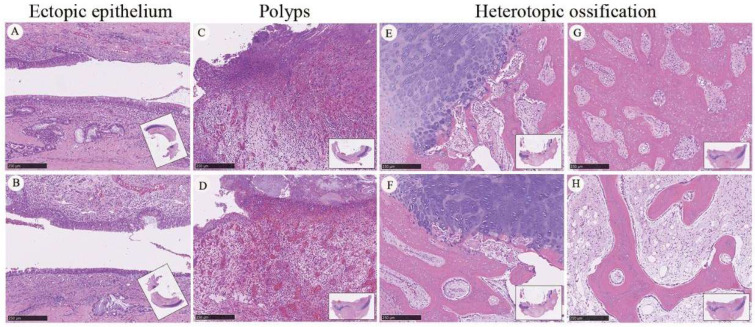
Pathological remodeling coincided with postoperative infection following two cm tracheal graft implantation. Representative histological findings from a two-cm bioprinted PCL tracheal graft harvested 12 weeks after implantation in an animal that experienced postoperative wound infection requiring surgical debridement. (**A**,**B**) Sections showing ectopic epithelial growth within the regenerated tissue, characterized by disorganized epithelial proliferation outside the normal luminal lining. (**C**,**D**) Polypoid lesions protruding into the graft lumen, coincided with localized inflammatory remodeling and altered epithelial–stromal architecture. (**E**–**H**) Areas of heterotopic ossification within regenerated cartilaginous regions, demonstrating aberrant mineralized tissue formation and disrupted cartilage architecture. These pathological features highlight abnormal tissue remodeling patterns that may arise under conditions of impaired infection control and secretion clearance during the postoperative period. Scale bars: 100 µm.

**Figure 8:**
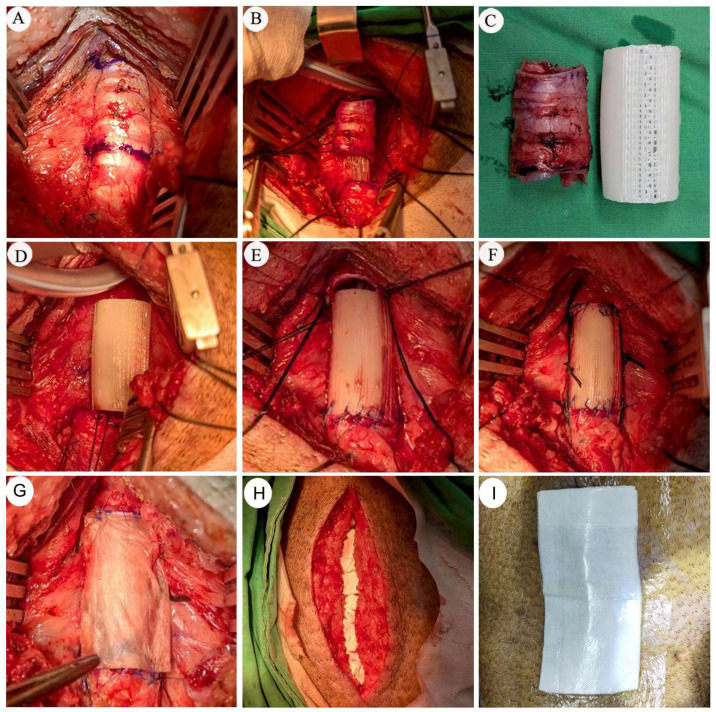
Intraoperative procedures and adjunctive wound management during long-segment (four cm) tracheal graft transplantation in a Lanyu porcine model. (**A**–**C**) Identification, marking, and circumferential resection of a 4 cm native tracheal segment. (**D**–**F**) End-to-end anastomosis of a bio-printed four-cm PCL tracheal graft at the proximal and distal interfaces, demonstrating satisfactory alignment and immediate structural stability. (**G**–**I**) Application of collagen- and growth factor-based biomaterials to the operative field for wound coverage and postoperative support, replacing conventional drainage devices to promote healing and reduce the risk of surgical site infection.

Spatial–temporal tissue regeneration within the established two cm bioprinted PCL tracheal graft was examined by gross examination and histological analysis. At 12 weeks after implantation, the reconstructed airway appeared to maintain luminal continuity and showed structural integration with adjacent native tracheal segments (Figure 3A). Cross-sectional sampling along the graft length revealed heterogeneous but organized tissue structures distributed across the proximal, middle, and distal regions of the implanted segment (Figure 3B,C-1–C-8).

Histological assessment using hematoxylin and eosin (H&E) staining demonstrated spatial variation in tissue architecture along the graft. More advanced tissue organization appeared to be present near the proximal and distal anastomotic regions, whereas comparatively less organized architecture was observed within the central portion of the graft (Figure 4). This spatial gradient may reflect differences in regenerative progression along the graft length, with more advanced organization observed near regions adjacent to native tracheal tissue and vascularized interfaces (Figure 4A). At the tissue level, lineage-specific differentiation patterns were observed during the regenerative process. Cartilaginous neotissue containing chondrocytes embedded within lacunae-like structures was observed by 12 weeks after implantation; however, the overall cartilage architecture remained less compact and less organized than that observed in native trachea (Figure 4B). Adipose tissue and skeletal muscle–like structures were also identified within the grafted region, indicating the presence of mesenchymal tissue components during regeneration (Figure 4C,D). Glandular structures were occasionally observed at later time points, whereas early regenerated tissue lacked organized gland formation (Figure 4E). Epithelial regeneration was observed along the luminal surface of the graft by 12 weeks after implantation. The regenerated epithelium formed a continuous lining and exhibited a stratified epithelial appearance; however, epithelial thickness, cellular organization, and surface maturation remained less developed than those of native tracheal epithelium (Figure 4F). Fully organized pseudostratified architecture and consistent ciliated differentiation were not uniformly observed at this stage, suggesting an immature epithelial regenerative state at the observed time point.

To further contextualize the maturation status of regenerated tissues, graft-associated neotissue harvested at 12 weeks after implantation was qualitatively compared with age-matched native tracheal specimens (Figure 5). In regenerated cartilage regions, H&E staining showed relatively high cellular density, with chondrocytes distributed within lacunae-like structures and a less compact extracellular matrix compared with native cartilage (Figure 5A,B,E,F). Glandular structures were identifiable as clustered epithelial units embedded within the submucosal region but exhibited less uniform organization than those observed in native trachea (Figure 5C–G). Regenerated epithelial layers covering the luminal surface were continuous but irregular in thickness and contour, with uneven stratification and incomplete epithelial surface organization (Figure 5D,H).

Native tracheal specimens harvested from three-month-old animals demonstrated more organized tissue architecture across cartilage, glandular, and epithelial compartments, characterized by reduced cartilage cellular density, more uniform glandular distribution, and clearer epithelial stratification (Figure 5I–L). In six-month-old native tracheas, these features were further refined, with dense and homogeneous cartilage matrix, mature glandular architecture, and a thicker, more organized epithelial lining (Figure 5M–P). Across the experimental cohort, regenerated tissues harvested from animals without postoperative wound infection showed more consistent tissue organization and fewer pathological alterations compared with specimens from animals complicated by postoperative infection.

Overall, comparison across developmental stages indicates that regenerated tracheal tissue at 12 weeks corresponds to an intermediate stage of histological maturation relative to native trachea. Although multiple tissue components—including cartilage, glands, and epithelium—were concurrently regenerated within the two cm PCL graft, their structural organization remained less mature than that observed in three- and six-month-old native tracheal tissues. These findings indicate that the bioprinted two cm PCL tracheal graft is associated with coordinated multi-tissue regeneration in situ, while revealing a developmental delay in histological maturation compared with native airway tissue. These findings are based on newly acquired specimens, while quantitative staging criteria have been described in our prior publications.

### 3.4. Cartilage Maturation and Staged Chondrogenesis Within Graft-Associated Neotissue

Cartilage regeneration within graft-associated neotissue appeared to undergo progressive morphological changes over time, rather than forming uniformly, as observed by sequential histological and immunohistochemical analyses (Figure 6). At 12 weeks after implantation, H&E staining revealed distinct cartilage-like structures exhibiting varying degrees of structural organization across different regions of the graft. In early-stage chondrogenic regions, chondrocyte-like cells were sparsely distributed within lacunae-like structures, and extracellular matrix (ECM) deposition appeared limited and loosely organized (Figure 6A,B). During the intermediate stage, focal matrix condensation became apparent, accompanied by localized chondrocyte remodeling and the emergence of vascular canal (VC)–associated chondro-modulator structures, suggesting ongoing structural reorganization within the developing cartilage (Figure 6C). In later-stage regions, cartilage neotissue exhibited relatively increased matrix density and enlarged lacunae, with reduced cellular heterogeneity, which may reflect progression structural maturation of the tissue (Figure 6D).

These histomorphological features show similarities to patterns previously described in staged chondrogenesis model, in which early-stage cartilage shows co-occurrence with higher Sox9 expression and increased proliferative activity, as reflected by elevated proliferating cell nuclear antigen (PCNA)-positive cell ratios, whereas advancing maturation corresponds to declining Sox9 expression, reduced proliferation, and progressive ECM accumulation [31]. The qualitative cartilage features observed in the present graft-associated neotissue appear generally concordant with these previously reported patterns, supporting a descriptive interpretation of progressive cartilage maturation within the bioprinted PCL graft (Figure 6).

To further characterize cartilage matrix development, cartilage-specific histochemical staining was performed at 22 days after implantation. Safranin O/Fast Green and Alcian blue staining demonstrated heterogeneous glycosaminoglycan (GAG) intensity during early chondrogenesis, followed by more prominent and spatially distributed staining in later stages, indicating increasing ECM deposition within developing cartilage (Figure 6E–L). These staining patterns paralleled ECM deposition patterns observed by H&E staining. In contrast, type II collagen immunoreactivity was detectable from early stages and remained consistently expressed throughout cartilage development period, suggesting the presence of a hyaline cartilage-like molecular matrix within the regenerating tissue (Figure 6M–P). Sox9 immunostaining revealed relatively higher nuclear expression in early-stage cartilage regions, with a gradual reduction in signal intensity in regions exhibiting more advanced structural organization, which may reflect progression of chondrogenic process (Figure 6Q–T).

### 3.5. Pathological Remodeling Coincided with Postoperative Infection in Tracheal Graft

Pathological tissue remodeling observed in association with postoperative infection were identified in a subset of animals undergoing two cm bioprinted tracheal graft implantation. During the initial phase of platform establishment, three LYD porcines received two cm tracheal grafts with placement of conventional surgical drainage tubes. Survival durations in this cohort were four weeks (*n* = 1) and 12 weeks (*n* = 2). Postoperative wound infection occurred in two of the three LYD animals. Following refinement of postoperative wound management protocols, three Lanyu porcines were enrolled in subsequent experiments. In this cohort, two animals underwent two-cm tracheal graft implantation with survival to 12 weeks, and one animal underwent exploratory implantation of a four cm tracheal graft with survival to two weeks. In all Lanyu animals, collagen-based biomaterials were used in place of conventional drainage tubes for postoperative wound coverage. No postoperative wound infection was observed in this cohort. Given differences in breed, experimental phase, and wound management strategy, these findings should be interpreted descriptively and not as evidence of a causal relationship between infection and the observed pathological features.

In one LYD animal that developed postoperative wound infection following two cm tracheal graft implantation, the histological examination of regenerated tissue harvested at 12 weeks revealed several abnormal histological features not observed in animals with uncomplicated postoperative courses (Figure 7). Sections from the grafted airway demonstrated irregular epithelial architecture along the luminal surface. Specifically, areas of ectopic epithelial proliferation were identified within the regenerated tissue, characterized by irregular epithelial stratification and extension of epithelial structures beyond the expected luminal boundary (Figure 7A,B).

In addition, polypoid tissue formations were observed protruding into the graft lumen. These polyp-like structures consisted of hypercellular tissue with inflammatory cell infiltration and altered stromal organization, resulting in partial luminal narrowing (Figure 7C,D). Marked pathological changes were also present within the regenerated cartilage. Foci of heterotopic ossification were identified in multiple regions of the cartilaginous neotissue, characterized by dense eosinophilic, bone-like structures replacing or interspersed within the cartilage matrix (Figure 7E–H). Such ossification was not observed in animals without postoperative infection and was distinct from the cartilage morphology observed in the established two cm graft model under stable postoperative conditions.

These observations suggest an association between postoperative infection and abnormal tissue remodeling, including ectopic epithelial growth, intraluminal polyp formation, and heterotopic ossification of regenerated cartilage. These alterations were identified in infected cases during the observation period, although the present study design does not permit causal inference. The histological features described here provide descriptive observations that may inform further investigation of infection-related complications and airway clearance challenges in graft-based tracheal reconstruction.

### 3.6. Exploratory Evaluation of Long-Segment (Four cm) Tracheal Graft Implantation

Exploratory transplantation of long-segment (four cm) bio-printed PCL tracheal grafts was performed to evaluate surgical feasibility and early postoperative airway observations in a large-animal Lanyu porcine model. This long-segment implantation was performed in a single animal (*n* = 1) and is reported as a preliminary exploratory observation. The four cm grafts were fabricated using the established printing parameters and implanted via circumferential tracheal resection followed by end-to-end anastomosis, extending the surgical framework validated in the two cm model.

Intraoperatively, a four cm tracheal segment was successfully identified, marked, and resected, followed by precise alignment and reconstruction using the PCL graft (Figure 8A–C). End-to-end anastomosis was achieved at both proximal and distal interfaces without gross mismatch or torsion, indicating that long-segment reconstruction could be technically performed under direct visualization (Figure 8D–F). To support postoperative wound healing and reduce the risk of surgical site infection, collagen- and growth factor-based biomaterials were applied to the operative field, replacing conventional drainage devices (Figure 8G–I). This adjunctive strategy was used as part of the refined wound management protocol described above during the early postoperative phase.

Early postoperative bronchoscopic examinations demonstrated initial airway patency immediately after surgery, with intact anastomoses and no evidence of graft displacement or tracheal collapse in the early postoperative period. One week after implantation, bronchoscopic surveillance revealed proliferative granulation tissue formation at the anastomotic regions, resulting in partial luminal narrowing. Targeted endoscopic laser ablation was, therefore, performed to relieve obstruction and restore airway caliber. Following intervention, temporary improvement in luminal patency was observed. However, prior to the scheduled second-week bronchoscopic follow up, the animal experienced unexpected sudden death during the night. Postmortem bronchoscopic examination revealed accumulation of blood clots and airway secretions within the distal grafted segment. Although a definitive cause of death could not be established, acute airway obstruction related to intraluminal clot formation and secretion retention was considered a possible contributor factor.

These observations indicate that four cm graft implantation can be technically performed, although maintenance of airway patency during the early healing phase appears vulnerable to granulation tissue formation and secretion retention. These findings highlight the importance of early airway surveillance and secretion management in extended tracheal reconstruction. Accordingly, outcomes from this case are reported descriptively, without inference regarding long-term survival or regeneration efficacy. They instead provide preliminary observations that may inform further refinement of surgical technique and postoperative airway management strategies.

## 4. Discussion

Currently, no definitive treatment exists for long-segment tracheal stenosis following extensive tracheal resection, regardless of malignant or benign etiology. Tracheal stenosis most commonly arises from iatrogenic injuries such as prolonged intubation, tracheostomy, and airway surgery, as well as trauma, chronic inflammation, and congenital anomalies. In addition, patients with pulmonary tuberculosis frequently develop long-segment tracheal stenosis as a result of airway involvement and post-infectious fibrotic remodeling, representing a clinically challenging subgroup with limited reconstructive options. Approximately one percent of patients undergoing tracheostomy develop clinically significant stenosis, a prevalence that has increased in the post-COVID-19 era [36]. Although the acute phase of the COVID-19 pandemic has passed, a substantial proportion of affected patients experienced severe respiratory failure requiring prolonged endotracheal intubation. This clinical course may contribute to an increased incidence of post-intubation tracheal stenosis in the post-pandemic period, thereby further highlighting the clinical relevance of tracheal reconstruction and, in severe cases, the need for tracheal transplantation.

Recent advances in 3D bioprinting have enabled the fabrication of airway scaffolds with controlled geometry, tunable mechanical properties, and reproducible architectural features, offering new possibilities for tracheal tissue engineering. Compared with conventional scaffold fabrication techniques, bioprinting allows relatively precise control over filament deposition and internal porosity, which is particularly relevant for mechanically dynamic organs such as the trachea. Despite these advantages and the expanding range of preclinical applications across multiple tissue types [37,38,39,40,41,42,43], translation toward clinically applicable airway reconstruction remains limited by challenges related to manufacturing scalability, biomechanical compatibility, and the coordination of long-term tissue regeneration [44,45,46,47,48,49,50,51]. The present study descriptively examines how fabrication strategy, surgical stabilization, and postoperative airway management may be associated with survival, regeneration, and complication profiles in a large-animal tracheal model.

### 4.1. Fabrication Strategy and Manufacturing Constraints as Determinants of In Vivo Performance

Manufacturing constraints intrinsic to extrusion-based 3D bioprinting appear to define practical boundary conditions for in vivo application. Short-segment (two cm) PCL grafts were fabricated with consistent structural configuration (Figure 1A–C), whereas long-segment constructs (≥four cm) approached operational limits of the printing system (Figure 1D–F). Increased build height was associated with minor filament deviations and reduced structural tolerance, reflecting cumulative mechanical constraints (Figure 1E,F). While these deviations did not preclude graft fabrication, they suggest practical engineering constraints for long-segment constructs and emphasize that build height, printing orientation, and toolpath stability may become increasingly important as graft length increases.

Accordingly, scaffold design, printing orientation, and segment length may influence biomechanical robustness and regenerative consistency when extending bioprinted tracheal grafts toward clinically relevant long-segment applications [52]. From a methodological perspective, these findings further support the central role of additive bioprinting in tracheal tissue engineering. Additive manufacturing uniquely enables controlled spatial architecture and, where applicable, incorporation of bioactive matrices during fabrication. In contrast, subtractive approaches such as laser machining or milling may improve surface finish or airflow characteristics but cannot readily embed biological cues within the construct during production. Hybrid strategies combining additive bioprinting with selective subtractive refinement may represent a potential engineering approach for long-segment tracheal reconstruction [53,54]. In the present large-animal study, no exogenous cells were incorporated into the PCL scaffold. This decision was deliberate. Surface-seeded cells on thermoplastic PCL constructs showed substantial loss after implantation in preliminary observations, likely due to mechanical stress and airway exposure. Therefore, we first aimed to determine whether the scaffold material and structural design alone could support host-derived tissue ingrowth. Establishing intrinsic regenerative compatibility was considered an initial step before introducing additional biological variables. Future studies will explore collagen surface coating combined with cell seeding and short-term in vitro preconditioning to enhance early cell retention and mucosal regeneration in long-segment reconstruction.

### 4.2. Integrated Survival Framework and Translational Wound Management Strategies

In the present porcine model, animals within the established two cm graft platform were able to maintain airway continuity during the observation period under a coordinated framework of mechanical stabilization, active postoperative airway intervention, and control of local biological conditions (Figure 2). Anchoring the graft to bilateral strap muscles helped maintain airway alignment and may mitigate anastomotic stress (Figure 2A–C). Serial bronchoscopic evaluation enabled early identification of granulation tissue formation, supporting timely laser ablation before critical luminal compromise (Figure 2D–F). Across experimental phases, differing wound management approaches were observed alongside differing infection frequencies. In earlier experiments incorporating conventional drainage tubes, postoperative wound infection was observed in a subset of animals, whereas in later experiments using collagen-based wound coverage materials, no overt surgical site infection was observed during the observation period. Given difference in breed, experimental phase, and perioperative management, these findings should be interpreted descriptively and without inference of causality.

Collagen- and growth factor–based extracellular matrices are widely used in reconstructive surgery and may offer theoretical advantages by reducing foreign-body exposure, supporting absorption of postoperative exudate, and offering a bioactive scaffold for soft tissue protection. Although the present study was not designed as a controlled comparison, optimization of wound coverage may represent an area for further investigation in scaffold-based airway reconstruction.

### 4.3. Spatial–Temporal Characteristics of In Situ Regeneration and Delayed Epithelial Maturation

The present findings suggest that tissue regeneration within the two-cm bioprinted graft occurs in a spatially heterogeneous manner rather than through uniform tissue replacement. Organized neotissue formation was observed along the graft (Figure 3), but regenerative progression consistently favored regions near native tracheal interfaces, whereas the central graft segment remained relatively less organized (Figure 4). This pattern may reflect the influence of local vascular supply and proximity to native tracheal tissue on regenerative progression [55,56,57,58,59,60,61].

Comparison with age-matched native tracheas demonstrated that multiple tissue compartments—including cartilage, glands, and epithelium—were regenerated but remained at an intermediate maturation stage (Figure 5A–H vs. Figure 5I–P). Notably, epithelial maturation appeared delayed relative to mesenchymal differentiation. Although continuous epithelial coverage was present, mature airway features such as consistent pseudostratified organization and ciliated differentiation were not uniformly achieved (Figure 5D,H). This dissociation between epithelial coverage and functional maturation may contribute to impaired mucociliary clearance during the early regeneration, potentially predisposing the reconstructed airway to secretion retention and secondary inflammation.

### 4.4. Length-Dependent Outcomes and Translational Context of Bioprinted Tracheal Grafts

Comparison between the established two cm platform and the exploratory four cm implantation suggests that increasing graft length may introduce additional biological and biomechanical challenges (Figure 8). While the two cm model supported sustained airway patency with coordinated multi-tissue regeneration under an integrated survival framework (Figure 4), extension to a four cm segment—despite technically successful resection and end-to-end reconstruction—exposed early vulnerabilities during the postoperative healing period (Figure 8A–F). In the single exploratory long-segment case (*n* = 1), serial bronchoscopic surveillance demonstrated preserved airway patency immediately after implantation but subsequently revealed rapid granulation tissue formation at the anastomotic regions, necessitating endoscopic intervention. Unexpected sudden death occurred prior to the scheduled second-week follow up. Postmortem bronchoscopy demonstrated accumulation of blood clots and airway secretions within the distal grafted segment. Although a definitive cause of death could not be established, acute airway obstruction related to clot formation and secretion retention may represent a possible contributing factor. These observations suggest that strategies effective for short-segment reconstruction may not be directly extrapolated to longer graft configurations without further optimization of airway clearance, granulation control, and epithelial maturation.

Previous studies in rodents, rabbits, sheep, and porcine models have demonstrated epithelialization and cartilage formation in bioprinted tracheal scaffolds; however, outcomes remain highly variable, particularly in large-animal models that more accurately recapitulate human airway biomechanics, immune responses, and postoperative management demands [34,56,57,58,59,60,61,62,63,64,65,66]. Within this context, the present porcine platform provides descriptive large-animal observations of scaffold-supported tissue regeneration while also highlighting unresolved translational barriers, including excessive granulation tissue formation, secretion retention, pathological differentiation, and heterotopic ossification.

### 4.5. Limitations and Future Directions

This study is limited by the small number of animals and the exploratory nature of the four cm implantation, for which long-term survival endpoints have not yet been achieved. The four cm long-segment transplantation was performed in a single animal (*n* = 1), and therefore conclusions regarding survival or regeneration efficacy cannot be drawn. In addition, observed differences in infection frequency across experimental phases should be interpreted cautiously, as the study was not designed as a controlled comparison.

Future work should prioritize the prevention of excessive granulation tissue formation, enhancement of epithelial functional maturation, and rigorous infection and secretion control. Long-term functional evaluation beyond airway patency—including biomechanical stability and prevention of pathological remodeling such as ectopic epithelial growth, polyp formation, and heterotopic ossification (Figure 7)—will be important for further evaluation of translational safety and long-term feasibility particularly for long-segment applications.

## 5. Conclusions

In the present large-animal study, two-cm bioprinted biodegradable PCL tracheal grafts were associated with survival beyond three months and progressive multi-tissue regeneration when combined with mechanical stabilization and structured postoperative airway management. In contrast, long-segment (four cm) transplantation was evaluated in a single exploratory case, and therefore conclusions regarding long-term survival or regenerative efficacy cannot be drawn. However, complete degradation of the PCL scaffold has not yet been achieved, and the degradation kinetics of PCL within the tracheal microenvironment remain incompletely characterized, which currently limits definitive assessment of the structural and functional integrity of fully regenerated tracheal tissue after long-term implantation. Addressing this limitation, together with challenges related to increased graft length, airway biomechanics, and epithelial maturation, will be important for further preclinical investigation and optimization prior to potential clinical translation, particularly for long-segment airway reconstruction.

## 6. Patents

The authors declare that there are no patents associated with the work reported in this manuscript.

## Data Availability

All data generated or analyzed during this study are included in this published article.

## Abbreviations

The following abbreviations are used in this manuscript:

3D	three-dimensional
PCL	polycaprolactone
CAD	computer-aided design
SIS	small intestinal submucosa
H&E	hematoxylin and eosin
ECM	extracellular matrix
VC	vascular canal
PCNA	proliferating cell nuclear antigen
GAG	glycosaminoglycan
